# They Saw It Coming: Rising Trends in Depression, Anxiety, and Suicidality in Creative Students and Potential Impact of the COVID-19 Crisis

**DOI:** 10.3389/fpsyg.2021.611838

**Published:** 2021-03-01

**Authors:** Barbara A. Kerr, Maxwell Birdnow, Jonathan Daniel Wright, Sara Fiene

**Affiliations:** Department of Educational Psychology, University of Kansas, Lawrence, KS, United States

**Keywords:** creativity & innovation, adolescence, anxiety, depression, COVID-19

## Abstract

Previous research has established that creative adolescents are generally low in neuroticism and as well-adjusted as their peers. From 2006 to 2013, data from cohorts of creative adolescents attending a counseling laboratory supported these results. Clinical findings of increased anxiety, depression, and suicidality among creative students in 2014 led the researchers to create 3 studies to explore these clinical findings. Once artifactual causes of these changes were ruled out, a quantitative study was conducted. Study 1, an analysis of mean differences of pre-2014 and post-2014 cohorts showed that post-2014 cohorts scored significantly higher in Neuroticism, Openness to Experience, and Conscientiousness and lower in Extraversion on Big 5 inventories. Regression analyses suggested that while Neuroticism was associated with gender, Conscientiousness and Grade Point Average for the earlier group, Neuroticism in the post 2014 groups was related to complex interplay of all personality dynamics except Agreeableness. In the qualitative Study 2, focus groups of 6–10 students, for a total of 102 participants were queried about the reasons they perceived for increased anxiety and depression in creative students. Increased achievement pressures and awareness of environmental and social problems were major sources of external stressors; perfectionism and desire to fulfill expectations of others were the primary sources of internal stress. The authors suggest that creative students' openness to experience and advanced knowledge made it possible for these students to see the potential for environmental and social crises and respond to their inability to solve these problems with anxiety and depression. Study 3 was a qualitative study that followed up 19 participants from the post-2014 cohort to explore the impact of the COVID-19 pandemic on mental health and creativity. While the majority perceived a negative effect of the pandemic on their mental health, most also produced a surprising variety of creative works during that time. In conclusion, rapid changes in the lives of creative adolescents since 2014 suggest that scholars focus on current cohorts and the ways in which adolescent personality is shaped by internal expectation and external pressures and global events. Despite the pandemic, creative young people continued to create.

## Introduction

The scientist-practitioner model of training has long been the ideal in clinical and counseling psychology training programs, with science informing practice, and practice stimulating research questions (Mallinckrodt et al., 2014). Most of the major ideas in the field of psychotherapy have arisen out of direct therapeutic contact, not from research directly (Beutler et al., 1995). Our study is an example of research that was prompted by anomalous clinical observations by doctoral counseling psychology students working in a research-through-service career counseling program for creative adolescents (the Counseling Laboratory for the Exploration of Optimal States-CLEOS). We sought to understand the anomalous findings beginning in 2014 of an increase in clinically observed and psychometrically assessed anxiety, depression, and suicidality in creative adolescents.

Since 2005, collaborating teachers at 28 schools have used profiles of trait complexes found to be associated with potential for creative productivity in 5 domains in order to identify potential innovators for our career counseling program. The profiles included high demonstrated ability in a creative domain (fine arts, performing arts, scientific and technology invention, creative writing, and interpersonal) as well as general creative traits, interests, and values; the profiles had been validated (Kerr and McKay, 2013) for predicting creative personality and creative productivity. Kerr and McKay (2013) found descriptions of clusters of domain-linked traits of fine and performing artists/writers and scholars/scientists similar to previous studies (Ivcevic and Mayer, 2006) and a large cluster of interpersonal creative traits similar to creative leadership studies (Reiter-Palmon and Illies, 2004).

Over the course of 12 years, students were selected through profiling and attended 1-day workshops in groups of 8–12. They took vocational and personality assessments, received individual interpretations, and engaged in group discussions and exercises designed to help them set goals and discover potential creative career pathways. Over that time, Students' personality traits revealed by the NEO- PI-R and NEO-PI-3 (Costa and McCrae, 1992) remained consistent. The modal profile of creative adolescents included high Openness to Experience, low Conscientiousness, and average scores in Neuroticism, Extraversion, and Agreeableness (Kerr and Vuyk, 2013). In counseling interviews, creative students were generally described by their counselors as congenial, engaged, and at low risk for depression, anxiety, and suicidal ideation. The number of preventive suicide assessments, triggered by a student's mentioning of suicidal ideation, was between 1 and 2 per year, with cohorts ranging from 46 to 140 students.

In the 2014 cohort, the levels of anxiety and depression were reported by students to counselors, and scores for Neuroticism on the NEO-PI-3 appeared to be higher than in the past. Out of the 42 students to participate in the first semester of the 2014 cohort, 14 required suicide assessment, preventive counseling, and/or referral. These unusual findings not only led to deep concern among therapists, but also the need to understand if this was a random occurrence or the beginning of a trend.

It was important to consider the possibility that these students were a sample drawn from a different population than the previous cohorts. First, we examined the schools that sent students to see if there had been a sudden change in the types of schools sending students. In 2012, 1 school with 20% of students receiving free and low-cost lunch, serving a university town, with higher than average statewide achievement test scores dropped out of the program and was replaced by a school with similar characteristics in all of the aforementioned factors except achievement test scores, which were somewhat higher. One school serving low-income rural students, with a majority of students on free and reduced lunch, dropped out and was not replaced. We checked the gender, income, and racial and ethnic makeup frequencies of visiting students and saw that the percentage of females and minority students was somewhat higher than in previous cohorts, and that they were slightly younger. The profiles, instructions to teachers, and nomination procedures had not changed; even 8 of 10 teacher nominators were the same. The forms of NEO-PI-R and NEO-3 instruments did not yield different scores, and the timing of changes in use of instruments did not correspond to changes in traits observed.

Several other explanations for the findings were explored, including a possible change in the training of counselors or the criteria for suicide assessment. Syllabi for courses in diagnosis and ethics were examined, with the finding that the same protocols had been taught for the last 6 years. What else could account for the sudden increase in depressed, anxious, and suicidal students?

Because these efforts did not yield an artifactual explanation for the observed increases in depression, anxiety, and suicidality, we developed 2 research projects, one quantitative and one qualitative to explore questions that would reveal information that might help us to understand and assist creative students in crisis. When the COVID-19 pandemic began in March of 2020, it was clear that we also needed to consider how this global crisis was affecting the mental health of creative students. A third project was initiated to follow up former students with an assessment designed to provide answers to the latter question. This paper will include the preliminary findings from that ongoing project.

The following research questions informed our investigation:

1. Are the trends observed in depression, anxiety, and suicidality of creative adolescents in our sample after 2014 paralleled by national trends in the prevalence of these disorders?
2. Can clinically observed changes in anxiety, depression, and suicidality of creative adolescents' from pre- and post-2014 cohorts be linked to changes in their modal Big 5 profile?
3. Are demographic, academic achievement, personality, and/or vocational interest variables available in the data including Gender, Age, Grade Point Average (GPA), other scales of the Big 5 personality tests, and scores on the 6 scales of the Vocational Personality Inventory associated with changes in the profiles?
4. How do creative adolescents in our sample explain increases in depression and anxiety in their own words, as inferred from their responses to focus group prompts concerning these topics?
5. How does a follow-up sample of former CLEOS participants describe their mental health and creativity after 10 months of the pandemic?

To attempt to answer these questions, we reviewed the literature on anxiety, depression, and suicidality in creative individuals and anxiety, depression, and suicidality in the general population of adolescents; compared personality profiles from early years of the program to later years; and attempted to predict anxiety and depression from our existing demographic, achievement, and personality data. We then conducted a pilot study using focus groups with the 2017 through 2019 cohorts of students to ask their own ideas about why anxiety, depression, and suicidality were increasing in creative adolescents. Finally, we followed up with 18 students who had attended CLEOS to explore the impact of the pandemic on their personality profiles, their experiences of depression and anxiety, and their experiences of creativity.

## Review of the Literature

Many creativity researchers have observed that the question, “Is creativity linked to mental illness?” is simply too broad (Silvia and Kaufman, 2010; Silvia and Kimbrel, 2010; Carson, 2019). Meta-analyses of studies of creativity and mental illness clarify that the answer varies depending upon research approach (Taylor, 2017); the types of psychopathology being investigated (Acar and Runco, 2012; Baas et al., 2016); the definition of creativity used, the measures used for both creativity and mental illness, the age at which creativity is measured, and the level of creativity of the participants (Paek et al., 2016). In general, effect sizes related to the link between creativity and mental illness are small; larger effect sizes tend to be found with specific subscales of measures; and stronger relationships tend to be found only in studies with adult eminent people and bipolar spectrum disorder, particularly in writers. Kaufman (2014) concluded, based on the positions of a wide variety of researchers, that the idea of a link between creativity and mental illness had been oversold to the public, and that research does not support the relationship. Instead, he said that many aspects of the creative process and creative life may be positive for mental health.

Studying milder pathologies seems more relevant to non-clinical samples of adolescents than the literature that focuses on severe pathology such as schizophrenia and bipolar spectrum disorders, which presents ambiguous or negative findings for creative individuals. It seems more fruitful to explore conditions such as anxiety and depression in creative adolescents. As Silvia and Kimbrel (2010) noted, anxiety and depression are by far the most common mental health problems, and yet are under-investigated in the literature of creativity and mental illness. They recommended that researchers consider the personality variable of Neuroticism, one of the Big 5 personality factors which represents a broad disposition to experience negative emotional states (Costa and McCrae, 1999).

High scores on measures of the Neuroticism factor are positively correlated with clinical and subclinical manifestations of mood disorders. Although meta-analyses of the relationship of Neuroticism to mental health disorders have shown moderate to high effect sizes for its predictive relationship to a wide range of diagnoses of mental illness, Neuroticism is less predictive of substance abuse disorders, narcissistic, antisocial, and paranoid personality disorders (Saulsman and Page, 2004). It is, therefore, not a comprehensive measure of psychopathology, but rather a predictor of the most commonly occurring mental health problems. Neuroticism is consistently negatively correlated with high ability and creativity (Furnham and Bachtiar, 2008; Batey et al., 2009; Clark and DeYoung, 2014). Batey and Furnham (2006), in a review of personality traits and creativity, found that Openness and Extraversion predicted divergent thinking, everyday creative behaviors, and self-reported creativity; but they found only a few examples of Neuroticism as a predictor of creativity, mainly within fine arts domains.

It seems, therefore, that neuroticism has no relationship, or an inverse relationship to creativity; that anxiety and depression are rare in carefully sampled studies; and that generally, creative students should be expected to be fairly well-adjusted.

## Adolescent Trends in Anxiety and Depression

The U.S. adolescent population in general appears to be increasingly anxious, depressed, and suicidal. In a nationwide study by the Substance Abuse and Mental Health Services Administration (SAMHSA, 2019), respondents were defined as having had a Major Depressive Disorder (MDD) in the past 12 months if they had at least one Major Depressive Episode (MDE), defined as a period of 2 weeks or longer in the past year when they experienced a depressed mood or loss of interest or pleasure in daily activities, accompanied by problems with sleeping, eating, energy, concentration, or self-worth. Adolescents were considered to have significant impairment if their depression caused severe problems with their ability to do chores at home, do well at work or school, get along with their family, or have a social life. In 2018, about 14% of adolescents aged 12 to 17 had a past year MDE and 10% had a past year MDE with significant impairment. The percentage of adolescents aged 12 to 17 in 2018 who had a past year MDE was higher than the percentages in 2004 to 2017. According to the results of the Pew Research Survey of Adolescents (Pew Research Center, 2019), 70% of teens saw depression, anxiety, and suicidality as major problems among their peers. While females were more likely than males to experience high levels of anxiety and depression, concern about mental health of peers cuts across gender, racial, and socio-economic lines, according to the study. Teens across demographic groups said it is a significant issue among their peers in their community. Fewer teens than in previous years were concerned about bullying, drug addiction, and alcohol consumption. More than 40%, however, said these were problems affecting people their age in the area where they live.

When it comes to the pressures teens face, academics are the major concerns: 61% of teens say they feel a lot of pressure to get good grades. By comparison, about 3 in 10 say they feel a lot of pressure to look good (29%) and to fit in socially (28%), while roughly 1 in 5 feel similarly pressured to be involved in extracurricular activities and to be good at sports (21% each). While about half of teens see drug addiction and alcohol consumption as major problems among people their age, fewer than 1 in 10 say they personally feel a lot of pressure to use drugs (4%) or to drink alcohol (6%) (Pew Research Center, 2019).

Adolescents may also turn to other maladaptive coping mechanisms. Non-suicidal self-injury (NSSI) rose by 8.4% each year for female adolescents from 2001 to 2015. This trend was particularly striking for girls aged 10–14, whose NSSI increased an average of 18.8% each year from 2009 to 2015. Female adolescents age 15–19 exhibited a similar, though less extreme, trend, with an increase of 7.2% each year from 2008 to 2015 (Mercado et al., 2017). In a study of data collected by the Centers for Disease Control and Preventions' National Vital Statistics System, Curtin and Heron (2019) found that youth suicide has steadily increased from 2007 to 2017. From 2000 to 2007, suicide rates in persons aged 10–14 dropped; they then tripled from 2007 to 2017. Similarly, suicide rates in persons aged 15–19 were stable from 2000 to 2007 and then increased by 76% from 2007 to 2017. Female adolescents (particularly age 10–14) are closing the previously sizable gap between male and female adolescent suicide (Ruch et al., 2019). Interestingly, suicide attempts and resulting injuries decreased from 1991 to 2017 for all racial groups except Black adolescents. Both Black girls and Black boys have shown increases in suicide attempts since 1991, and Black boys are significantly more likely to experience injury from a suicide attempt (Lindsey et al., 2019).

Perhaps no other contributor to the literature on the trend of rising anxiety, depression, and suicidality among adolescents has had more public influence than Twenge (2020), who compared these trends to a number of other social trends and concluded that the explanation was increased use of technology. The mechanisms, she said, included displacement of time spent on in-person social interactions as cultural norms evolve. These trends were seen both individually and across the generations. Additional mechanisms identified were interference with sleep time and quality, cyberbullying and toxic online environments, and online contagion and information about self-harm. What is extraordinary about Twenge's conclusions is that she dismissed other explanations such as rising inequality (it had been going on since the 1980's, she claimed) and ignored other obvious trends: racism, homelessness, environmental destruction, failing schools, and the rising costs of college (Henriksen and Mishra, 2020). Only recently has the connection between teens' use of technology and mental disorders been submitted to rigorous analysis.

Odgers and Jensen (2020) comprehensively reviewed these studies and found that most research was correlational, focused on adults vs. adolescents, and generated a mix of conflicting small positive, negative, and null associations. Instead, they said, the most recent and rigorous large-scale pre-registered studies reported only small associations between the amount of daily digital technology usage and adolescents' well-being. Without a way of distinguishing cause from effect, they explained, these findings are unlikely to be of clinical or practical significance.

The changes in creative adolescents that we had observed appeared to mirror those of the United States adolescent population.

## Study 1: Comparison of Big Five Personality Profiles of Pre- and Post-2014 Cohorts of Creative Students and Analysis of Relationship of Gender, Age, GPA, and Vocational Interests to Neuroticism

### Participants

Participants in this study were 2 cohorts of adolescents who had been identified by gifted education coordinators of 28 Midwestern high schools as matching the profiles of eminent people when they were adolescents in 5 domains (Art and Design; Music; Writing; Invention; Interpersonal Creativity). We used all of the students' data from those that had complete files. The 2006–2013 cohort included 131 adolescents out of 167 participants who completed all instruments (64 female, 64 male, 3 non-binary) who attended in groups of 10- 12. The later cohort included 170 out of 265 adolescents who completed all instruments, and who attended, also in small groups, from 2014 to 2019 (83 female, 80 male, 5 non-binary, 2 not stated). Their race and ethnicity were similar in both groups, and mirrored the proportions of the area from which they were drawn: 85% (2006–2013) and 84% (2014–2019) European-American; 9% (2006–2013) and 6% Asian or South Asian-American; 3% (2006–2013) and 4% (2014–2019)African-American; 2% (2006–2013) and 2% (2014–2019) Latine/Hispanic-American, 1% (2006–2013) and 1% (2014–2019) Native American. They averaged 16.3 years of age in early cohort and 16.1 in the later cohort.

### Instruments

The instruments included a demographic self-report form developed for the purpose of the project, the Vocational Preference Inventory, and the NEO-PI-r and the NEO-PI 3.

All students took the Vocational Preference Inventory (VPI; Holland, 1996). The VPI is a 160-item measure appropriate for individuals from 14 to 75 years old. The VPI is one of the most widely used vocational interest tests with adolescents and young adults and consists entirely of occupational titles; patterns of responding to these titles yield 3 letter codes representing the individual's vocational personality. Factor analyses of the VPI have consistently yielded 6 clusters of vocational personalities, including Realistic, Investigative, Artistic, Social, Enterprising, and Conventional, and the predictive, construct, and concurrent validity are well-established, as well and as all forms of reliability. Additionally, the VPI was found to be correlated to assessments with similar scales, such as the Strong Vocational Interest Blank (Gaffey and Walsh, 1974).

Although there are a few competing models of personality, the Big 5 Model of personality remains the dominant 1 after 4 decades, valid across cultures and predictive of a wide variety of valued human behaviors (Feher and Vernon, 2020). Of the Big 5 personality inventories, the NEO- PI-R and the NEO-PI 3 are considered as highly reliable and valid measures of personality. The 2006–2013 cohorts of students took the NEO-PI-R (Costa and McCrae, 1992). The NEO-PI-R is a well-established measure that yields 5 dimensions of personality and is appropriate for older adolescents (Soto et al., 2008). This 240-item assessment has coefficient *alphas* of 0.92 (Neuroticism), 0.89 (Extraversion), 0.87 (Openness), 0.86 (Agreeableness), and 0.90 (Conscientiousness). Numerous studies have supported the construct validity and reliability of the instrument.

The 2014–2018 cohorts of students took the NEO-PI-3 (McCrae et al., 2005). The earlier version had been found to have 30 items that were difficult for some adolescents. The authors used self-report and observer rating data from 500 respondents aged 14 to 20 to select replacement items. The modified instrument retained the intended factor structure and high reliability and validity, and showed better internal consistency, cross-observer agreement, and readability. Scores on both the NEO-PI-r and NEO-PI-3 were standardized (Costa and McCrae, 1992; McCrae et al., 2005) by using T-scores to allow for comparison across the 2 cohorts, as recommended by McCrae et al. (2005).

The factor Neuroticism typically includes 6 sub-facets of anxiety, depression, vulnerability, anger-hostility, impulsiveness, and self-consciousness; it is generally characterized as a sensitivity to threat and punishment and is considered indicative of maladjustment.

The factor Openness to Experience is the personality factor characterized by inventiveness, curiosity, and an appreciation for a variety of experiences. It is the personality variable most often associated with creativity. Facets of openness to experience include fantasy, feelings, ideas, aesthetic sensitivity, actions, and values (Costa and McCrae, 1992). Openness to Experience, according to Vuyk et al. (2016a), is a stronger construct than the commonly used construct of “overexcitabilities” to explain creatively gifted adolescents' heightened sensitivity and imagination.

Extraversion is the factor associated with outgoingness and leadership. Extraversion is described as sociable, assertive, active and talkative, cheerful, upbeat, energetic and optimistic (Costa and McCrae, 1992).

Agreeableness is defined as kind, sympathetic, warm, cooperative, and considerate, and it encompasses the facets of trust, straightforwardness, altruism, compliance, modesty, and tender-mindedness (Costa and McCrae, 1992).

Conscientiousness is the personality factor most often associated with academic success and is a strong predictor of academic performance (O'Connor and Paunonen, 2007). Conscientiousness is described as a person's tendency to plan, be goal-directed, delay gratification, and adhere to social norms and rules. Conscientiousness facets include achievement striving, competence, deliberation, dutifulness, order, and self-discipline (Costa and McCrae, 1992). For this study, the scores for the 5 factors of NEO-PI-R and the NEO-PI-3 were standardized so that scores could be compared for both cohorts on Neuroticism, Openness, Extraversion, Agreeableness, and Conscientiousness.

### Analysis

*T*-tests were performed on scores for all Big 5 personality factors for 2006–2013 and 2014–2019 cohorts in order to determine if the personality profiles of creative students differed for the 2 cohorts. In addition, *t*-tests were performed on Holland vocational interests, gender, age, and Grade Point Average (GPA). Hierarchical multiple regression analysis is used to identify if the variables of interest explain a significant amount of the variance in the dependent variable. This test, therefore, was used to determine the amount of variance demographic, academic, interest, and the other personality variables contributed to the prediction of Neuroticism. Regressions were not performed on other Big 5 Factors at this time, because the focus of this study was the mental health of the creative adolescents, which is best captured by Neuroticism. By including other Big 5 personality factors as predictors, it was possible to explore how observed changes in personality profiles might have contributed to Neuroticism. Because the Big 5 factors are not completely orthogonal, and because overlap has been found with the Holland vocational interest factors, an analysis of multicollinearity was performed. All variables except one were in the proper range (VIF < 2) except the Extraversion/Enterprising pair, for which the VIF value was 2.0.

### Results

Table 1 shows the descriptive statistics for both groups and the results of the *t*-tests of differences between means for the 2006–2013 and 2014–2019 cohorts on each of the Big 5 factors. The 170 participants who attended the CLEOS program in the 2014–2019 cohort (*M* = 59.94, *SD* = 30.47) compared to the 131 participants in the 2006–2013 cohort (*M* = 49.96, *SD* = 10.32) had significantly higher Neuroticism scores, *t*_(199)_ = 3.57, *p* < 0.001. The effect size was small to medium, Cohen's *d* = 0.44. The participants in the 2014–2019 cohort (*M* = 35.82, *SD* = 31.32) compared to the participants in the 2006–2013 cohort (*M* = 49.13, *SD* = 11.05) had significantly lower Extraversion scores, [*t*_(199)_ = 5.54, *p* < 0.001]. The effect size was medium to high. Cohen's *d* = 0.57. The participants who attended the CLEOS program in the 2014-2019 cohort (*M* = 50.62, *SD* = 11.42) had higher Openness to Experience scores compared to the participants in the 2006–2013 (*M* = 77.95, *SD* = 20.62) and (*M* = 55.38, *SD* = 11.52), *t*_(199)_ = 5.16, *p* < 0.001. The effect size was small to medium, Cohen's *d* = 0.39. The participants who attended the CLEOS program in the 2014–2019 cohort (*M* = 54.65, *SD* = 30.39) had significantly higher Conscientiousness scores than the earlier cohort (*M* = 47.35, *SD* = 10.97), *t*_(199)_ −5.43, *p* < 0.001. The effect size, Cohen's *d* = 0.32 was small to medium. Agreeableness was the only factor that had no differences found in mean scores.

**Table 1:** Descriptive statistics and independent samples tests for big five, holland codes, gender, age, and GPA of early (2006–2013) and late cohorts (2014–2018).

	**Early cohort**			**Late cohort**				
	**Mean**	***SD***	***N***	**Mean**	***SD***	***N***	***t***	***p***
Openness to experience	55.38	11.52	131	77.95	20.62	170	5.16	0.00^*^
Conscientiousness	47.35	10.97	131	54.65	30.39	170	−5.43	0.01^**^
Agreeableness	51.95	14.18	131	54.04	32.45	170	−0.15	0.88
Extraversion	49.13	11.05	131	35.82	31.32	170	−3.97	0.01^**^
Neuroticism	49.96	10.32	131	59.94	30.47	170	−3.43	0.00^*^
Realistic	2.91	3.19	131	4.13	3.51	170	0.18	0.01^**^
Investigative	5.89	4.09	131	7.40	4.05	170	−1.10	0.01^**^
Artistic	6.77	4.51	131	7.02	4.53	170	−1.25	0.35
Social	4.53	3.41	131	5.14	3.96	170	−0.05	0.06
Enterprising	3.19	3.31	131	4.12	3.75	170	0.27	0.01^**^
Conventional	1.37	2.11	131	2.25	2.93	170	0.92	0.01^**^
Gender	1.51	0.50	131	1.60	0.54	170	4.31	0.01^**^
Age	16.29	1.00	131	16.08	1.18	170	−2.08	0.05^*^
GPA	3.70	0.51	131	3.72	0.36	170	−3.43	0.01^**^

*NEO-PI Big 5 Personality Traits are Neuroticism, Extraversion, Openness to Experience, Agreeableness, Conscientiousness*.

*Holland Vocational Interests are Investigative, Artistic, Social, Enterprising, Conventional, and Realistic*.

*GPA, Grade point average*.

* *p < 0.05 level*.

** *p < 0.01 level*.

For the Holland vocational interests, Students in the later cohort scored higher on Realistic (*M* = 4.13, *SD* = 3.51) than the earlier cohort, (*M* = 2.91, *SD* = 3.19), *t*_(199)_ =0.18, *p* < 0.01, Cohen's *d* = 0.36, a low to medium effect size. Students in the later cohort scored higher on Enterprising (*M* = 4.12, *SD* = 3.75) compared to those in the earlier cohort (*M* = 3.19, *SD* = 3.31), *t*_(199)_ =0.27, *p* < 0.01 and Cohen's *d* = 0.26, a low effect size. The later cohort also scored higher on Conventional, *M* = 2.25, *SD* = 2.93 compared to the earlier group (*M* = 1.37, *SD* = 2.11), *t*_(199)_ =0.92, Cohen's *d* = 0.35, a low to medium effect size. They scored higher in Investigative (*M* = 7.40, *SD* = 4.05) than the earlier group as well (*M* = 5.89, *SD* = 4.09), *t*_(199)_ = 1.10, *p* < 0.0, Cohen's *d* = 0.37, a low to medium effect size. The demographics of the groups were different, but all of the differences had low or very low effect sizes. There were more females in the later cohort (*M* = 1.60, *SD* =0.54) than in the earlier cohort (*M* = 1.51, *SD* =0.50), *t*_(199)_ = 4.31, *p* < 0.01, Cohen's *d* = 0.17, a low effect size. The later group was younger, (*M* = 16.08, *SD* = 1.18) than the earlier group, (*M* = 16.29, *SD* = 1.00), *t*_(199)_ = −2.08, *p* < 0.05, Cohen's *d* = 0.19, a low effect size. The later group had a slightly higher GPA, (*M* = 3.72, SD = 0.36) compared to earlier students (*M* = 3.70, *SD* = 0.51) *t*_(199)_ = −3.43, *p* < 0.01, with a Cohen's *d* = 0.04, a very low effect size.

Table 2 shows the results of the regression analyses for 2006–2013 and 2014–2019 cohorts for Neuroticism, in which gender, age, Grade Point Average, vocational interests (RIASEC), and the other 5 factors were predictors. Just as the 2 groups had different personality profiles, they also differed in the variables that were predictive of neuroticism. For the 2006–2013 cohort, the regression was significant. Results of the linear regression indicated that there was a collective significant effect of the gender, Conscientiousness, and GPA [*F*_(12,118)_ = 3.34, *p* < 0.001, *R*^2^ = 0.25). The individual predictors were examined further and indicated that only the status of being female was positively associated with Neuroticism, while Conscientiousness, and GPA were negatively associated with Neuroticism.

**Table 2:** Regression analysis coefficients for neuroticism of vocational interests, personality traits, gender, age, and GPA of 2006–2013 (*n* = 131) and 2014–2019 (*n* = 170) cohorts.

	**2006–2013 Cohort**					**2014–2019 Cohort**				
**Variable**	**B**	***SE***	***Beta***	**t**	***p***	**B**	***SE***	***Beta***	**t**	***p***
(Constant)	61.04	16.17		3.77	<0.001^*^	27.88	43.7		0.64	0.52
Investigative	−0.24	0.243	−0.10	−0.99	0.32	−0.22	0.64	−0.03	−0.34	0.74
Artistic	−0.35	0.26	−0.15	−1.3	0.18	1.02	0.62	0.15	1.63	0.11
Social	−0.08	0.2	−0.02	−0.26	0.80	0.98	0.73	0.13	1.34	0.18
Enterprising	0.14	0.32	0.05	0.45	0.66	−0.76	0.83	−0.09	−0.91	0.36
Conventional	0.41	0.44	0.08	0.91	0.36	−0.17	0.94	−0.02	−0.18	0.86
Realistic	0.003	0.30	0.001	0.01	0.99	−1.28	0.80	−0.15	−1.6	0.11
Openness to experience	0.13	0.01	0.14	1.31	0.19	0.28	0.13	0.19	2.22	0.03^*^
Conscientiousness	−0.26	0.09	−0.28	−2.9	<0.001^*^	−0.07	0.08	−0.07	−0.86	0.39
Extraversion	−0.10	0.09	−0.11	−1.2	0.25	−0.19	0.08	−0.20	2.54	0.01^**^
Agreeableness	−0.08	0.06	−0.10	−1.2	0.23	−0.08	0.06	−0.10	−1.2	0.23
Gender	8.56	1.99	0.42	4.29	<0.001^*^	1.53	4.66	0.03	0.33	0.75
Age	0.31	0.87	0.03	0.35	0.72	0.09	1.96	0.003	0.05	0.97
GPA	−4.18	1.92	−0.21	−2.1	<0.01^*^	4.07	6.46	0.05	0.63	0.53

*GPA, Grade point average*.

*Holland Vocational Interests are Investigative, Artistic, Social, Enterprising, Conventional, and Realistic*.

*NEO-PI Personality Traits are Extraversion, Openness to Experience, Agreeableness, Conscientiousness*.

* *p < 0.05 level*.

** *p < 0.01*.

For the 2014–2019 cohort, the regression was also significant, *F*_(12,157)_ = 3.76, *R*^2^ = 22. Openness to experience positively related to Neuroticism, and Extraversion was negatively related. Vocational interests were not predictive of neuroticism for either cohort. Ackerman and Heggestad (1997) performed a meta-analysis of Big 5 personality traits and Holland vocational interests and found considerable overlap of traits and interests, holding that interests explain preferences for particular environments and traits explain motivations and styles within environments. Although multicollinearity measures were acceptable, results of the present study included high correlations in both groups for Artistic interests and Openness to Experience (0.43 and 0.57) and moderately high correlations of Enterprising and Extraversion (0.38 and 0.26); this may have led to the personality traits overwhelming the variance due to vocational interests. Additional discussion of how the dynamics of profile change may have contributed to increases in Neuroticism will be found in the concluding discussion.

## Study 2: Creativity and Rising Neuroticism: A Multiple Focus Group Study

### Method

Adolescent CLEOS participants in the 2017 through 2019 cohorts were invited to participate in a focus group during their original visit to CLEOS and all agreed to do so. The purpose of this focus group was to directly ask creative adolescents what they believe are the causes of the trend of increasing depression, anxiety, and suicidality observed in creative adolescents in 2014 and the following years. Data for this study was derived from the 2017 and 2018 focus group cohorts (14 groups*; n* = 109). Adolescent clients were invited to take part in the focus group during the afternoon following their individual career counseling sessions. Larger groups of participants were separated into two separate focus groups based on gender and non-binary students were invited to join either group. Students and facilitator(s) sat together in a circle and the facilitator passed out a sheet labeled “Causes” on one side and “Solutions” on the other, with lines for students to list the collective themes agreed upon by the group. Participants were given a brief introduction to the purpose of the study and the methods of the focus group. They were also informed that the focus group would be recorded for transcription purposes.

The method used for the discussion is variously called cumulative voting, sticky-dot voting, or votocracy (Hill et al., 2009). It is a method developed for prioritizing issues and allowing participants to express the strength of their preference as well as their ranking of preferences. Long used by youth groups such as 4-H and civic organizations, it is considered a way of hearing all voices and allowing the strength of convictions as well as clarifying which of issues are perceived as most important. The facilitator of the focus group posed the first question for verbal discussion: What do you think is causing this rise in depression and anxiety among creative adolescents? Participants were encouraged to share their ideas with the group and to respond to each other's ideas. Less talkative participants were specifically encouraged to share their ideas if they had not yet done so. Before each theme was established, participants were asked to agree on a concise name for the theme as a group. The presence of a theme on the list reflected a general consensus that each theme might be a cause of the trend in question. The facilitator periodically asked participants if any of the themes should be subsumed under a broader theme and students typically reached a consensus in this case as well.

Once causal themes were established, participants were asked to write down each theme on the “Causes” side of their paper. The facilitator then instructed each participant to distribute 10 dots (drawn with pencil, pen, crayon, or dry-erase marker; or using stickers) among the themes on the paper, assigning more dots to the causes they saw as more impactful to the trend of rising anxiety and depression. They were instructed to distribute the dots in any way they chose that represented the power with which they held their opinion of the importance each theme (e.g., 10 on 1 theme, or 5 on 2 themes, or 3 on 3 themes and 1 on 1 theme, etc., adding up to ten).

Audio from the focus groups was transcribed by the investigators and was analyzed for content by masters-level graduate students in counseling psychology who had served as CLEOS counselors. Due to audio issues, 4 of the session recordings were not available for transcription. Each analyst was provided an explanation of the procedure for the focus group, the transcript for one of the recorded groups, and the list of causal themes identified by the respective group. Analysts were asked to highlight passages of the transcript that represent the details of each overarching theme identified during the corresponding focus group. In addition, coders identified causes that seemed psychological or biological in origin, vs. causes that seemed to be related to external stressors. This data was then entered into a spreadsheet. The 4 experimenters then examined once again the ranked causes and solutions of each group, compared this raw data to the analysts' highlighted passages, and categorized each of the themes identified during focus groups into study-level categories.

### Results

All study-level categories of causes identified by focus group participants, as well as the number of votes for the themes in each category, are listed in Tables 3, 4 below. Table 3 shows the frequency of themes that represent external pressures and stressors identified by focus groups, while Table 4 shows the frequency of themes that represent intrapsychic and individual factors that contribute to increasing Neuroticism. Thirty-six external thematic categories and 17 intrapsychic/individual thematic categories were identified. The most endorsed thematic category across all focus groups with 133 total votes was “Higher Expectations/Pressure,” which refers to the extra pressure that creatively gifted students feel to achieve greater and greater things. Once they meet high expectations, they are often met with even greater expectations and feel like they must be adept at everything they attempt. Many participants agree with the notion that the “average excellence is increasing,” meaning the bar has risen for talented individuals, leading to often “impossible” expectations put on them by valued others (e.g., teachers, parents, siblings, college admissions agents). This is closely related to the intrapsychic thematic category of “Perfectionism/Fear of Failure,” which had the second most amount of votes across all focus groups (81 total votes). Sometimes creatively gifted students center their identity around achievement (“I have to be good, I have to be better. Or what am I?”; “If I can't do it then I'm letting myself and everyone else down”). Perfectionism may lead to physical health issues (“You can either choose perfection or physical health. You choose perfection each time”) and isolation.

**Table 3:** External causes of rising neuroticism.

**Item**	***n***	**% of votes**
Higher Expectations/pressure	133	22.7
Being different	63	10.7
Social media	60	10.2
Mental health awareness	37	6.3
School structure/system	28	4.8
Technology-age of info	27	4.6
“Invisible” problems/“imaginary” problems	22	3.7
Lack of recognition	19	3.2
Parental support	16	2.7
Increased stress at school	16	2.7
A lot of competition	14	2.4
Underestimation	13	2.2
Lack of freedom/expression	11	1.9
Generational differences	11	1.9
Limited friendships	8	1.4
Immediate gratification	8	1.4
Doing better than previous generation	8	1.4
Lack of power	8	1.4
Lack of innovation	7	1.2
Changes in parenting styles	7	1.2
“You're so special”	7	1.2
Lack of community	7	1.2
Lack of acceptance of mental illness/stigma	7	1.2
No education about emotions/adulting	6	1.0
Undervalued education	6	1.0
Bias against gifted students	6	1.0
Division/choosing sides	5	0.9
Lack of social development	5	0.9
Poor school funding	4	0.7
Everything is intersectional	4	0.7
Lack of physical activity	3	0.5
World-Threatening problems	3	0.5
Post-911 cohort	3	0.5
More career possibilities	3	0.5
Government sad disease	1	0.2
Parental divorce	1	0.2

**Table 4:** Intrapsychic/individual causes of rising neuroticism.

**Item**	**n**	**% of votes**
Perfectionism/fear of failure	81	33.3
Higher awareness/involvement in important issues/politics	40	16.5
Inability to be authentic	20	8.2
Existential depression	18	7.4
“I don't know who i am”	13	5.3
High sense of responsibility	10	4.1
“Me mentality”/mean mentality	10	4.1
Victim mentality	9	3.7
Self-diagnosis	8	3.3
Fear of death	6	2.5
Overacting and overthinking	6	2.5
Priorities, not able to follow flow	6	2.5
Super active minds	5	2.1
Brain development	4	1.6
Non-help-seeking behaviors	3	1.2
Escape into digital media	2	0.8
Increased drug and alcohol use	2	0.8

Creative adolescents also report “Being Different” (63 total votes) and “Social Media” (60 total votes) as major contributors to the trend of rising Neuroticism in their population. They believed they were viewed as abnormal due to their differences from less creative students, and some believed that there are qualitative differences, such as higher empathy and sympathy than the general population. Creative adolescents may feel ostracized because they believe if peers see them as having an advantage in something, those peers may feel disadvantaged and hold hostility toward creative adolescents. Participants note that social media has the potential to magnify these differences and their effects as well as the potential to help them find communities of like-minded individuals. Importantly, participants were by and large ambivalent about social media. They discussed it as a tool that can be used for connection, marketing, expression, and other positive endeavors. Many of them were “digital creatives” (Hoffmann et al., 2016) whose area of creativity was technology; but they also discussed social media as a place to make damaging upward comparisons between themselves and others, especially those who share their creative interests, and a place to be constantly inundated with negative media.

The theme “Higher Awareness/Involvement in Important Issues/Politics” is the fifth-highest rated theme across focus groups with 40 votes, and the second highest intrapsychic/individual cause. Conceptually related by some groups to the perceived higher capacity for empathy of creative adolescents, many participants noted that they feel more aware of current events than their less creative peers. This greater awareness of societal and world events, such as environmental destruction and political polarization, seems to be associated with a sense of foreboding and dread. Many also endorsed that they experience the emotions surrounding these events more deeply than their less creative peers, leading to more negative thoughts and feelings. This is of particular interest when considering how creative adolescents may respond to the COVID-19 pandemic. Taken in context with the aforementioned top themes, creatively gifted adolescents may be at high risk for setting unattainable goals in the context of the pandemic while simultaneously experiencing the emotional effects of isolation and fear of illness and death with more gravity than their less creative peers.

## Study 3: How the COVID-19 Pandemic Influences the Mental Health and Creative Output of Creative Young Adults

Study 3 was designed to answer two primary questions. The first question was “How has the COVID-19 pandemic impacted the mental health of creative young adults?” The second was “Have young adults previously identified as creative been engaging in creative endeavors during the ongoing COVID-19 pandemic?” To answer these questions, former clients of the Counseling Laboratory for the Exploration of Optimal States (CLEOS) responded to open-ended questions via an online survey which investigators then analyzed using a qualitative open coding procedure. Participants were also asked to complete a Big 5 measure of personality to help contextualize open-ended responses and compare potential differences in response to the pandemic based on personality factors. This is an ongoing follow-up project, and these findings are preliminary. All but one of the respondents are part of the post-2014 cohort.

### Participants

The investigator located potential participants from a database of individuals who previously participated as clients in the CLEOS Project. Participants had previously been identified by faculty at their school as being creatively gifted in one or more categories. In total, 18 participants took part in this study (*N* = 18). Fourteen participants were White (77.8%), and there was 1 participant from each of the following 4 racial/ethnic categories (5.6% each): Black/African-American, Asian/Asian-American, Middle-Eastern, and Multiracial. Ten participants (55.6%) were female; interestingly, the remaining 44.4% of participants was divided evenly among those who were male (*n* = 4) and those who disclosed non-binary genders (*n* = 4). Participants had an average age of 20.2; 22.2% of participants (*n* = 4) were age 19 and 4 participants were also age 20 (22.2%). Three participants (16.7%) were age 18 and 3 were age nineteen. Two participants (11.1%) were age 22. One participant was 23 and 1 participant was 24 (5.6% each). All but one of the participants were members of the Post-2014 cohort.

### Instruments

#### 50-Item IPIP Version of the Big 5 Markers

A 50-item Big 5 personality inventory available through the International Personality Item Pool (IPIP) was used to measure participants' Neuroticism, Extraversion, Openness to Experience, Agreeableness, and Conscientiousness (International Personality Item Pool, 2019, 2021). There are ten questions for each of the 5 factors. Neuroticism is measured as “Emotional Stability” (ES), meaning lower scores on this factor indicate higher Neuroticism. Scores on each factor range from zero to forty, and Neuroticism scores are calculated by subtracting participants' scores on Emotional Stability from forty. This inventory was first developed by Goldberg (1992) in an attempt to develop markers for the Big 5 factor structure and it has since “been used by researchers in dozens of published studies” (International Personality Item Pool, 2019, p. 5). This brief inventory does not measure specific facets within each of the 5 Factors. Ehrhart et al. (2008) demonstrated good to excellent internal consistency reliability for each of the inventory's factors: E (α =0.89), A (α =0.78), C (α =0.81), ES (α =0.86), and O (α =0.78). These values were similar between men and women and between racial groups included in Ehrhart's sample (2008, White, Asian/Asian-American, and Latine). While these authors express the need for additional inquiry into the measure's construct and criterion validity, this measure provides an easily accessible and affordable alternative to larger measures of Big 5 personality factors, such as the NEO-PI-3. Although there are no adolescent norms, this instrument can help researchers to understand relative high and low scores.

##### Open-Ended Questions

Participants were asked the following open-ended questions designed to elicit responses to the 2 primary questions:

How have you been spending time during the COVID-19 pandemic? Work? School? Caring for Family? Other?
What creative work have you been doing since March?
What else can you tell us about how you have spent your time since March?
Many CLEOS students were dealing with significant anxiety and depression before the pandemic. How has the COVID-19 pandemic affected your mental health?
What would people close to you say about your mental health during the pandemic?
During your CLEOS visit, we did an exercise in which we asked you to visualize your Perfect Future Day. What do you remember from your vision of the future? What does your vision of the future look like now?

Questions regarding participants' Perfect Future Day visualizations were included to elicit responses that may illustrate how COVID-19 has disrupted or reinforced participants' visions of the future. Additionally, this question was included to study the lasting impact the activity has on former CLEOS clients.

##### Demographics Questionnaire

Participants were asked to self-describe their race/ethnicity and gender identity instead of checking the box that best represents these identities. Race was coded as either “White,” “Black/African American,” “Asian/Asian-American,” “Middle-Eastern,” “Latine,” or “Multiracial” and gender was coded as either “male,” “female,” or “non-binary.” Middle-Eastern and Asian/Asian-American were kept separate to subvert the pattern of erasure of Middle-Eastern identities in psychological literature, despite the “Middle East” existing largely in the Asian continent. Non-binary was used as an umbrella term for any participant identifying as a gender other than “male” or “female.” Participants were asked to report their age in years and asked if they consent to contact from CLEOS for further follow-up studies.

### Procedure

The investigator contacted potential participants via email and Facebook direct message to invite them to participate. This invitation included a link to the online survey hosted on the Qualtrics website. Participants were provided information about the study and asked to provide consent by clicking a checkbox to show their agreement and providing an e-signature using their mouse or touch screen. Once participants granted consent, they were asked to fill out the 50-item IPIP measure of Big 5 personality traits. Once they finished this measure, they were asked the open-ended questions listed in the Materials section. Then they filled out a demographics form, completing the survey, and were thanked for their time.

### Analysis

Analysis of qualitative survey data was performed using open coding and constant comparison procedures inspired by constructivist grounded theory (Thornberg and Charmaz, 2014). Open coding involves the researcher picking a unit of analysis (e.g., sentence) and coding every instance of that unit with themes, reflecting on the meaning of categories and their underlying processes. Codes are always considered provisional, and the researcher “compare[s] data with data, data with code, and code with code” (Thornberg and Charmaz, 2014, p. 158). Glaser and Strauss (1967), the founders of grounded theory, used these constant comparison analyses to assist in developing theory and improve the generalizability of findings. In this study, the unit of analysis is each case (i.e., the experience of each participant). This includes information conveyed through subunits of individual words, dyads of word, parts of compound sentences, and entire sentences, depending on the meaning conveyed by the subunit and its functionality within context.

The investigator first scored the 50-item IPIP measure of Big 5 personality traits for each participant in order to add a quantitative measure personality to the qualitative analysis procedure. These were added to a spreadsheet with participants' responses to the open-ended questions. The investigator then read through each set of open-ended responses, taking notes via paper and pen for each participant. This information was entered into a separate spreadsheet in the form of both lists and qualitative codes. List categories included Activities, Emotions, Cognitions, PFD Original, and PFD Now.

Deductively-generated qualitative codes followed the research questions and included Creative Activity (whether the participant had engaged in creative work since the start of the pandemic, yes or no); COVID-19 Mental Health Impact (coded as either Positive Impact, Negative Impact, or Neutral Impact); Mental Health Congruence (coded as Congruent, Incongruent-lower, or Incongruent- higher based on the participants' expectations of how valued others view their mental health during the pandemic); PFD Congruence (coded as 0, 1, or 2); and PFD Recall Detail. During the coding procedure for these deductively-generated codes, the investigator observed patterns of responses that were frequent enough to inductively generate open codes. These included Minimization of Engagement (the participant disclosed their creative engagement while simultaneously minimizing this engagement); Hides MH Issues (the participant explicitly disclosed that they hide their mental health struggles from valued others); Bleak Future (the participant explicitly disclosed a highly negative view of the future in general); Self-deprecation (the participant questions their talent or mental health in a pejorative way); COVID-19 Setback (the pandemic “took away” something from the participant, leading to more mental health issues); Religiosity (the participant's religion was noted as a major factor in their goals, mental health, or both); Sleep Increase (the participant disclosed sleeping more than normal during the pandemic, including increased overnight sleep and/or in the form of excessive naps), and; Extra Time/Slow Down (the participant disclosed that the pandemic gave them the time to recommit to valued activities and self-care). The investigator coded responses for these inductively-generated open codes.

In order to compare cases, the investigator took extensive notes on each case using a coding journal as he read through participants' responses. He compared cases to one another based on observed patterns of responses, which aided in developing open codes. After a provisional full set of open codes was developed, the investigator coded all cases and entered the coded data into a spreadsheet. He then used a random number generator to form 9 dyads of cases and compared the cases in each of these dyads to one another, again taking extensive notes and observing patterns of responding. This method of constant comparison allows themes to emerge that persist throughout the comparisons. Persistent themes included mostly negative effects of the pandemic on mental health (12 cases); continued creative (16 cases) or intellectual (2 cases) work; minimization or derogation of own creative work (12 cases); and fears about the future (12 cases).

### Results

The impact of COVID-19 on the mental health of this sample of creative young adults was by and large negative. Twelve of the 18 participants (66.7%) identified that the pandemic has had a negative impact on their mental health, with 4 (22.2%) saying that their mental health has improved since its onset (coded as Positive Impact and Negative Impact, respectively). Two participants (11.1%) noted no significant change to their health since the start of the pandemic (coded as Neutral Impact). Twelve participants (66.7%) disclosed that others who are close to them would agree with their mental health self-assessment.

The mean and standard deviation for each of the sample's Big 5 personality factor scores can be observed in Table 5. Out of a total score for 40 on each factor scale, the Study 3 sample scored an average of 32.78 on Openness to Experience (*SD* = 5.77); 22.5 on Conscientiousness (*SD* = 7.52); 15.72 on Extraversion (*SD* = 7.78); 31.83 on Agreeableness (*SD* = 6.35); and 26.56 on Neuroticism (*SD* = 8.96). While the sample size of Study 3 is too small to adequately compare participants' IPIP Big 5 scores to NEO scores from previous studies' cohorts, one can observe that Openness to Experience remains the highest score, followed by Agreeablenss, Neuroticism, Conscientiousness; Extraversion is the lowest score. The pattern of high Openness to Experience and low Extraversion match the later cohort from which most were drawn. The high Agreeableness score may reflect this sample's willingness to be interviewed. The somewhat low Conscientiousness score may reflect the participants' negative assessment of their productivity during the pandemic.

**Table 5:** Study 3 pandemic follow-up results on the 50-item IPIP measure of big 5 personality traits.

**Factor**	**Mean**	**SD**	**Minimum**	**Maximum**
Openness to experience	32.78	5.78	17	40
Conscientiousness	22.50	7.52	8	35
Extraversion	15.72	7.78	2	31
Agreeableness	31.83	6.35	18	40
Neuroticism	26.56	8.96	8	37

The majority of participants, nevertheless, have been engaged in creative activities during the pandemic (16; 88.9%). The investigator coded each of the activities in terms of both the O^*^NET designation and the Holland Code designation, as listed by the O^*^NET online catalog of career codes (National Center for O^*^NET Development, 2021). Please see Table 6 for a list of activities coded as creative. There were no significant differences between the groups regarding the detail with which they recalled their Perfect Future Day visualizations; similarly, there were no differences regarding the congruence of participants' original PFD visualizations with their current visions of the future and or life circumstances. Findings from the comparison analyses were used in the development of open codes and to prevent overgeneralization based on one specific case.

**Table 6:** Activities engaged in by participants since March 2020.

**Holland code**	O*NET vocation	**Activities**	***n***	**%**
AES	Musicians and singers	Playing cello	2	11.1
AI	Poets, lyricists, and creative writers	Creative writing (fiction and non-fiction), poetry	5	27.8
AI	Special effects artists and animators	Animation	1	5.6
AR	Fine artists, including painters, sculptors, and illustrators	Illustration, painting, general art	6	33.3
AR	Photographers	Photography	1	5.6
ARC	Cooks, private household	Cooking	2	11.1
ARE	Graphic designers	Digital art	1	5.6
CI	Social science research assistants	Psychological research	1	5.6
EAS	Producers and directors	Making tiktoks	1	5.6
ECR	Online merchants	Running online business	1	5.6
ICA	Mathematicians	Mathematics	1	5.6
IEA	Urban and regional planners	Writing (housing policy)	1	5.6
IR	Physicians, pathologists	Anatomical concept-mapping	1	5.6
IR	Soil and plant scientists	Taking care of plants	1	5.6
IRE	Agricultural engineers	Building a chicken coop	1	5.6
R	Painting, coating, and decorating workers	Decoration	1	5.6
RAC	Jewelers and precious stone and metal workers	Jewelry-making, metalsmithing	2	11.1
RAI	Musical instrument repairers and tuners	Making a hurdy-gurdy	1	5.6
RC	Construction laborers	House renovation	2	11.1
RC	Bakers	Baking	1	5.6
RCI	Carpenters	Carpentry	1	5.6
SAI	Philosophy and religion teachers, post-secondary	Collaborative writing (philosophy)	1	5.6
SIA	Mental health counselors	Connecting with others, building relationships	7	38.9
SRC	Personal care aides	Caring for family	3	16.7

*Holland codes include Realistic, Investigative, Artistic, Social, Enterprising, and Conventional as well as combinations thereof*.

O*NET Vocations were chosen based on their similarity to participants' creative endeavors in order to classify activities by Holland code.

*GPA, Grade point average*.

*Activities, those activities engaged in by participants since March 2020*.

*n, number of participants who engaged in each subset of activities*.

*%, percentage of sample who engaged in each subset of activities*.

#### Negative Impact

Twelve participants disclosed that the COVID-19 pandemic had a negative impact on their mental health. Four participants disclosed that valued others would misjudge the pandemic's impact on the participant as lower than it actually is (i.e., valued others would judge the participant as more mentally healthy than they actually are). Three of these 4 participants explicitly disclosed in their responses that they hide their mental health issues from valued others. One participant said that securing employment has partially abated the pandemic's negative impact; they were still coded as belonging to the Negative Impact group due to the initial and overall negative impact of the pandemic.

Five participants in this group disclosed feeling anxious in response to the pandemic, and 5 disclosed feeling isolated. Isolation here refers to a lack of sufficient interactions with others while loneliness refers to a lack of quality in relationships with others. Three disclosed feeling depressed. Other emotions disclosed by this group include distractedness, fear, grief, hope, optimism, lack of motivation, worry, angst, boredom, despair, distress, loneliness, passion, stress, and uncertainty. Some participants in this group noted that their negative reactions to non-pandemic issues have increased in frequency and/or valence thanks to the “backdrop” of COVID-19. One participant noted that her friends would describe her recent behavior as “unpredictable.”

Both participants who did not disclose engagement in creative activities since the beginning of the pandemic viewed the pandemic as negatively affecting their mental health, and both of them were experiencing academic transitions (one was beginning their college career online and the other beginning medical school). Some participants viewed their distress as stemming from a combination of anxiety about the pandemic and other life stressors, such as school, indicating life transitions may mediate the pandemic's effect on mental health.

Three participants in the Negative Impact group expressed, to varying degrees, the belief that the future is bleak. One explicitly attributed their bleak view of the future to the COVID-19 pandemic in combination with US politics, and another noted that “the world will end in 5 years” when asked about their current goals for the future. This reveals an underlying emotion to the experience of some participants in this group that was not explicitly disclosed: hopelessness. For these participants, visualizing their Perfect Future Day was a moot activity. Multiple participants in this group cite the pandemic as a source of anxiety due to its form of negative punishment; one lost what could be considered their dream job in the music industry and another had recently made life changes to improve her mental health that were undone by the pandemic. Two participants in this group noted that they have been sleeping more often than normal during the pandemic.

Of note, 3 of the participants in the Negative Impact group exhibited some form of self-deprecation in their responses. This came in the form of criticizing one's own creative work, using significantly negative language to refer to their mental health, or assuming their mental health struggles are attributable to their being a “hypochondriac.” Similarly, 2 participants minimized their creative engagement while also endorsing that they have been engaged in creative endeavors since the beginning of the pandemic with specific examples.

#### Positive Impact

All of the participants who felt the pandemic had a positive effect on their mental health disclosed that others who are close to them would agree with their mental health self-assessment. For example, 1 member of this group, who achieved eminence on TikTok during the pandemic, noted that others would say they are “glowing again.” Two of the 4 participants who disclosed a positive impact of the pandemic on their mental health noted changes in their relationship with time that facilitated this positive impact. These 2 participants noted that the world slowed down in response to COVID-19, allowing them to take the time to engage in meaningful activities. One of these two used this extra time to explicitly focus on their mental health, and the other used this time to “reconnect with nature.” Neither explicitly mentioned emotions. The other 2 participants who experienced a positive impact on their mental health still disclosed feelings of worry, sadness, and anxiety in their responses, and one expressed hope for the future. Interestingly, the average scores on each of the Big 5 personality factors was higher for the Positive Impact group than for the Negative Impact group. Of the 3 participants in this group who remember the content of their Perfect Future Day visualization, all of them still hold similar goals and visions for the future.

#### Neutral Impact

Both participants who disclosed experiencing no impact on their mental health due to the pandemic have been engaged in creative activities since its onset. One did not disclose how their mental health self-assessment aligned with valued others' perceptions; the other participant disclosed that valued others would say the pandemic had a more negative effect on their mental health than it actually did due to the participants' increase in sleep. Participants disclosed feelings of isolation, worry, and anxiety, sometimes vaguely (e.g., “I get bummed out sometimes with not being able to do and see everyone that I want to”). Both participants recalled the content of their Perfect Future Day visualizations, one in vivid detail, and both still had at least somewhat congruent current goals and plans for the future. Interestingly, one member of this group who scored lower than the sample mean on Openness to Experience disclosed attitudes and beliefs specifically demonstrating her Openness that stem from her relationship with God (i.e., following the opportunities presented to her by God as they arise).

## Discussion

In answer to our first question, “Are the trends observed in depression, anxiety, and suicidality of creative adolescents in our sample after 2014 paralleled by national trends?” we found clear parallels. Comparing the current studies of creative adolescents attending the CLEOS project to what is known about creativity and mental illness among creative adolescents, the students observed and assessed before 2014 were similar to research-based descriptions of creative adolescents in the literature (Silvia and Kimbrel, 2010; Kaufman, 2014). They were open to ideas, values, actions, aesthetics, and fantasy. They were average in Neuroticism, neither depressed, anxious, vulnerable, self-conscious, and angry nor overly optimistic, insensitive, incautious, unaware, or defenseless. In short, they were well-adjusted teens. Creative students after 2014 were significantly higher in Neuroticism, that is, more depressed and anxious, more vulnerable, more self-conscious, and more volatile. They were significantly less extraverted, so they were less warm and gregarious, less active, and less excitement-seeking in their orientation compared to the earlier group. They were significantly more conscientious, and therefore, more serious, industrious, orderly, achievement-oriented, and deliberate. Interestingly, despite their worried, vulnerable, inward orientation, they remained open to experience, scoring significantly higher than the earlier cohort, and were creative, imaginative, intellectual and open to ideas, values, and actions. In fact, despite their neurotic symptoms and strong conscientiousness, their t-scores showed them to be more than 2 standard deviations above adolescent norms on Openness. Yet, they were unlike any descriptions of creative adolescents in the literature.

The creative students in our study, however, were similar to adolescents in general in terms of their depression, anxiety, and suicidality, the rising trends of which were noted in major national surveys (SAMHSA, 2018; Pew Research Center, 2019). The only way in which they differed from adolescents in general is that they seemed to suffer from these disorders earlier than their less creative peers. Given the extraordinary increase in suicidality of our creative students in 2014, it might be said that they not only experienced these afflictions earlier, but also more intensely.

Our second question was, “Can clinically observed changes in anxiety, depression, and suicidality of creative adolescents' from pre- and post-2014 cohorts be linked to changes in their modal Big 5 profile?” Our post-2014 cohort had profoundly different profiles, with significant differences from the pre-2014 cohort on all scales except Agreeableness. They were less extraverted, more conscientious, and much more open to experience, in addition to having higher Neuroticism.

The third question we asked was, “Are any demographic, academic achievement, personality and/or vocational interest variables available in the data associated with changes in Neuroticism. In order to understand how their overall profiles might have been related to the observed increase in neurotic symptoms, we investigated, through regressions, potential predictors of Neuroticism scores for both cohorts. For the 2006–2013 cohort, the status of being female was associated with higher neuroticism; female adolescents were more likely than male adolescents to be experiencing neurotic symptoms such as anxiety, depression, vulnerability, and difficulty controlling emotions. Similar to the Pew Research Center (2019) findings, adolescents self-identified as female were more likely to be anxious and depressed than male adolescents. In addition, the lower the scores on conscientiousness, and the lower the student's GPA, the more likely the student was to experience symptoms of neuroticism. For the 2006–2013 cohort, where neuroticism was one of the lowest scores in the profile, it seems that those students who were struggling with their grades and lacking in the aspects of conscientiousness needed to improve their grades were more likely to be depressed and anxious. It makes sense that the only students experiencing mental health issues in a cohort that was for the most part very well-adjusted were those that were struggling with their grades. Vocational interests were not predictive of neuroticism for this group.

The profile of the second cohort was quite different from that of the first, and the predictors for neuroticism were also quite different. It may be that very high openness to feelings, ideas, and fantasy makes them more vulnerable to anxiety and depression when confronted with stressful and unprecedented events. Williams et al. (2009) found greater resilience under induced stress conditions for undergraduates with high openness to experience; however, samples of undergraduates such as were used in their study tend not to tap the population of those who are very high in openness to experience. On the other hand, DeYoung et al. (2012) found a “paradoxical simplex” in their study of Openness to Experience, showing that at the highest levels of the Openness aspect of Openness to Experience (as opposed to the Intellect aspect), people may experience instability and apophenia, the tendency to make unusual, non-practical associations of ideas. Reduced extraversion also may play a part in increased Neuroticism. Extraversion has long been found to be associated with resilience and well-being, predicting positive mental health after 40 years. (Gale et al., 2013). Being less extraverted, they may turn to others less for help and affirmation and turn more inward, reflective, and sensitive to stress. Neither demographics nor vocational interests were predictive of neuroticism for this group. Rather, it is the complex dynamics of the personality profile that provide the main clues to the diminished mental health of this group.

Our fourth question was, “How do creative adolescents in our sample explain increases in depression and anxiety in their own words, as inferred from their responses to focus group prompts concerning these topics?” The focus group study we conducted with creative adolescents provided a richer picture of their own perceptions of what was causing the upward trend of anxiety and depression. More than twice as many external causes were named as internal causes, indicating a tendency to look to the problems they saw in the world around them more than to seek intrapsychic, individual causation. Interestingly, the leading cause they perceived could be considered an interaction of external and internal causation; that is, the extreme pressure this group experienced to fulfill what often seemed impossible expectations of parents, schools, and society. Their own perfectionism and fear of failure inevitably led them to try to meet expectations that they be academically successful, involved in activities, and creative in all ways. Coupled with this was an awareness of the impossibility of achieving all of these goals in a society that no longer provided the support they needed and a world that had utterly changed from the ones their parents and teachers knew. In addition, these students felt that they were perceived by others as “being different.” The role of social media tended to be associated mainly with the exaggeration of their sense of themselves as outsiders. Few students saw rising use of technology in general as important to anxiety and depression, but rather saw the specific impact of the ways in which people misuse social media.

The second most common internal cause of distress was enhanced awareness of social, political, and global issues. Avid use of technology to learn more about the world may have indeed increased this distress; on the other hand, the increased sensitivity to, and knowledge about these issues coupled with little power to bring about change certainly contributed to despair for this group. External pressures from parents and teachers, and their internalized sense of responsibility to meet societal expectations collided with their awareness that they could not possibly address the enormity of the problems they saw around them, no matter how creative they might be.

Like canaries in a coal mine, these creative young people were signaling to the adults around them that something was very wrong, that disaster was coming, and that they were caged in by their youth and helpless to do anything about it. They did not specifically see the coming pandemic; but they saw the bigger picture of environmental destruction and political upheaval, and they called out to the adults around them to recognize their dread as real and to do something about it.

To some degree, the results of the second study affirmed the personality dynamics found to be associated with Neuroticism in the first study. The participants were aware of heightened awareness of stressful events in their lives and the world, as well as their strong response to stress. They clearly identified their desire to fulfill the expectations of their families and society while feeling frustrated by their lack of power to do so.

To answer our final question, “How does a follow-up sample of former CLEOS participants describe their mental health and creativity after 10 months of the pandemic?” we conducted a follow-up of 18 young adults, all but one of whom represented the post-2014 cohort. Our participants, like most young people, were profoundly affected by the pandemic. Life transitions, job loss, and social isolation compound creative young adults' distress similarly to how they do in the general population, leading to greater anxiety, depression, sleep, and hopelessness. Yet certain specific challenges, such as losing a dream job in a creative field due to the pandemic, may have a greater impact on these creative young adults who have specifically pursued personal meaning in their vocational experience. A minority of creative adults may actually flourish due to the interruption of mundane daily routine and overwhelming responsibilities. The Kaiser Family Foundation found that nearly 75% of all people felt their lives directly affected either some or a lot by coronavirus, and nearly 75% felt the worst was yet to come (Panchal et al., 2020). Forty percent of adult respondents suffered loss of job, loss of income, or reduction of hours and pay as a result of the crisis.

The experiences of young adults can provide insight into the experiences of adolescents. Similarly to the adults in our sample, teens have been directly affected by the pandemic, often because of the need to suddenly switch to online education, the need to assist in childcare, and for low-income families, the need to assist the family financially through work in low-paid jobs. Like adults, adolescents must now worry about potential infection and death, testing availability, treatment options, vaccine development, and the uncertainty about humanity's new normal.

In addition, adolescents have unique issues resulting from the pandemic. Social isolation, difficulty with online education, increased risk for domestic discord and violence, and difficulty seeking help for mental distress are some of the issues discussed by Fegert et al. (2020) in their narrative review of concerns for child psychiatrists and therapists.

Kuzujanakis (2020) described how the pandemic may have affected (and continues to affect) gifted students. Like many in gifted education, Kuzuianakis speculated that gifted students' heightened sensitivities and “overexcitabilities” could put them more at risk for mental health issues as a result of the pandemic. The belief in overexcitabilities in gifted students is not without its detractors, given that the construct of overexcitabilities is almost entirely overlapping with openness to experience (Vuyk et al., 2016b). The confounding of cognitive abilities with personality traits means that it is difficult to ascertain whether it is the high intellectual capacity or the increased openness to emotions, ideas, fantasy, aesthetics, values, and actions that might make students more sensitive. Given, however, that creative students' highest personality score on Big 5 Tests tends to be Openness to Experience, there may be value in considering the additional risk that world events pose for creative students.

## Limitations

A number of limitations must be considered in interpreting the data. First, despite the measures taken to rule out artifactual causes of differences between the pre-2014 cohort and the post-2014 cohort, such as ensuring the same schools used the same profiling methods to identify young people, there may have been other reasons for the abrupt change in the profiles of the creative students. We discussed many potential correlates with the increase in depression and anxiety, but none of the ones we discussed or described in popular media featured prominently in the focus group themes. In addition, small to moderate effect sizes require that the finding of different profiles should be interpreted with caution. Second, without a control group of less creative students, it is difficult to discern which changes were related to the students' creativity as opposed to changes related to experiences common to adolescents in the Midwest. It is important to consider, given the 2014–2019 cohorts extremely high Openness scores, that 8 years of practice in the profiling method had improved teachers' ability to identify truly creative students. Third, when regressions were done, all potential variables for which data was available were used, when, as it turned out, the large number of variables and the overlap of vocational interests and personality traits may have reduced the possibility of more predictors emerging. Fourth, the focus groups, by their very nature, addressed very specific questions that primed the students to focus on negative aspects of their lives, as opposed to more positive or hopeful statements.

Finally, one notable limitation to study 3 is the small sample size. Had more creative young adults participated, it may have been possible to compare their profiles as adolescents to their current profiles as young adults. The small sample size also limits the generalizability of these findings, as well as the occurrence of each open code; however, it is important to note that global catastrophes have the potential to impact everyone, and people within specific groups may experience similar psychological sequelae.

## Implications

With these limitations, implications must be drawn with caution. It seems, however, that we may need to consider carefully when we make generalizations about creative adolescents—or for that matter, adolescents in general—from data more than a decade old. With the rapid changes occurring globally, each new cohort of youth merit open-minded exploration of their attitudes, emotional well-being, and reactions to changes in their community and society. Second, clearly we need further exploration of the relationship of personality factors to one another, not just psychometrically, but also from the point of view of adolescents. How do they think low Extraversion, high Neuroticism, high Conscientiousness, and high Openness work together? Or more informally, how does one continue to be creative despite isolation, depression, anxiety, and conscientiousness?

Third, all of those who work with creative young people should be aware of the possible “canary in the coal mine” effect—that is, the sense of foreboding, the existential dread these young people experienced about the state of the world was not adolescent dramatizing. With their openness to ideas, sensitivity, and curiosity, they seemed to discern patterns of impending collapse while the adult world was concerned with other matters. They did not specifically foretell the pandemic, but they knew a global crisis was coming in their lifetimes.

Our strongest takeaway from these glimpses into the personalities, the inner thoughts and feelings, and the behaviors of these young people was a hopeful one. Despite the modesty with which our creative adolescents described their writing, art, music, and social activism, we saw a clear theme of the persistence of creativity in the face of inner turmoil and a global pandemic. We hope that as we continue the long term follow up of the CLEOS participants we will learn more about how these young people transcend personal difficulties and continue to create in a time of crisis.

## Data Availability Statement

The original contributions presented in the study are included in the article/supplementary material, further inquiries can be directed to the corresponding author/s.

## Ethics Statement

The studies involving human participants were reviewed and approved by University of Kansas Institutional Review Board. Written informed consent to participate in this study was provided by the participants' legal guardian/next of kin.

## Author Contributions

BK contributed original concept, original literature review, design of first study, conduct of first study, first section of write up, final revision, and editing. MB contributed to literature review, design and implementation of second study, analysis of second study, and write up of results for an APA poster session. JW participated in conceptualization, design, contributed to literature review, and analyzed data from first study for an APA poster session. SF participated in integration of studies in an APA poster session, data tables, and editing of review. All authors contributed to the article and approved the submitted version.

## Conflict of Interest

The authors declare that the research was conducted in the absence of any commercial or financial relationships that could be construed as a potential conflict of interest.
